# Patients and glaucoma: what are the challenges?

**Published:** 2012

**Authors:** Mohammed Abdull

**Figure:**
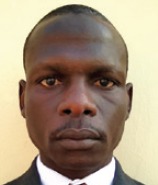
Mohammed Abdull

**In general, how advanced is the glaucoma in patients presenting at your hospital?**

Most of our patients present with advanced disease. The usual presentation is with one eye blind and the other with moderately advanced disease. We think this is because they are not aware of glaucoma and do not understand how important it is to seek help.

**How did you find out what people thought about glaucoma?**

We met with a number of patients’ relatives and traditional healers and conducted a series of interviews and focus group discussions. The term ‘glaucoma’ was not known by many people. Those who had heard the term had different ideas about what it meant. There is no local word for the disease. Some knew it as ‘black blindness’, as opposed to ‘white blindness’ (cataract).

Different people had different ideas about the causes of glaucoma. Some thought it was caused by excessive crying, excessive anger, or excessive exposure to firewood smoke. Others related it to black flies or confused it with river blindness. Some understood it as hypertension of the eye. Very few people related it to death of the nerve of the eye.

**What prevents people from coming forward early enough?**

The main reason is the lack of symptoms. This, together with poor awareness of the existence of the disease, means that people wait until the eye is blind or there is substantial loss of vision before they come.

Also, people in this area know about cataract and tend to wait until the cataract is mature before they come for an operation. So those experiencing some sight loss often assume they have cataract and by the time they come it is too late to save their sight.

**Figure:**
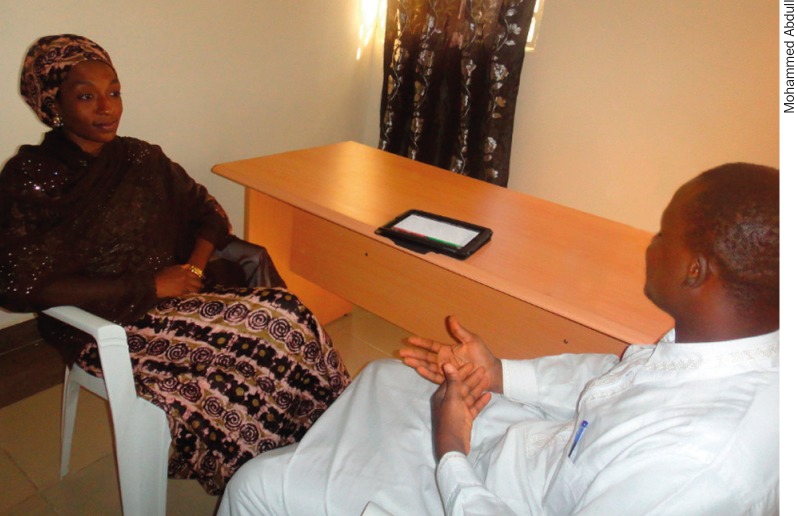
Good communication with patients is an important element of glaucoma care (see page 54). NIGERIA

People also waste a lot of time seeking alternative or traditional medication. This is because, in many communities, traditional healers are more easily available and accessible. They come to the patient's house and offer their services, which the patient is often unable to refuse because the healers are recommended by influential relatives and they may initially offer free service or service on credit.

Treatment provided by traditional healers can sometimes cause physical harm, but perhaps the most serious consequence is that this will cause a delay in patients seeking and receiving the correct medical treatment. The consequence of this delay is very often frustration for the health worker, grief for the family, and irreversible blindness for the patient.

**Figure:**
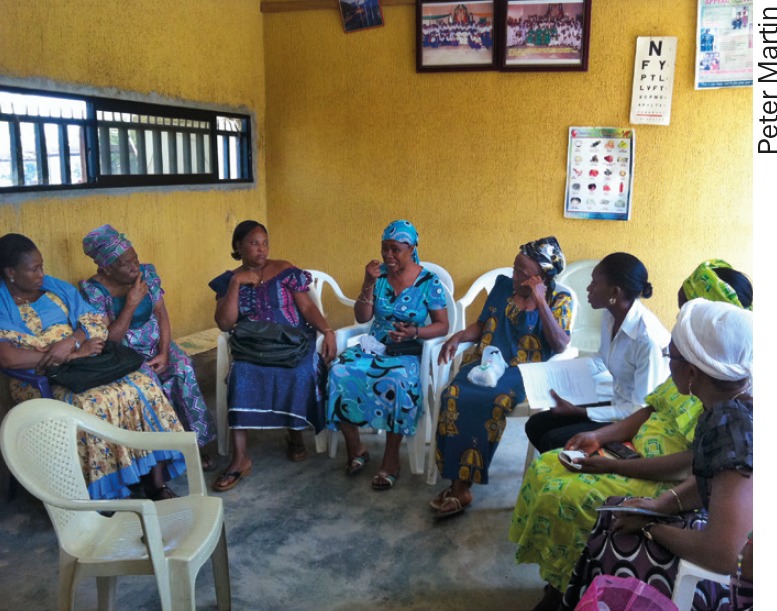
A focus group discussion about glaucoma. NIGERIA

**How can we encourage people to come forward early enough?**

I think the key to getting patients to treatment sooner is to create awareness, emphasising the lack of initial symptoms and urging the people most at risk to go for a check-up. These are people with first degree relatives with blindness or glaucoma, people wearing glasses for myopia, those on long-term steroid treatment, and all adults over 40 years with diabetes, hypertension, or a history of trauma to the eye.

Radio and television can make a big difference. Every time we talk about any disease on TV or radio, our clinics are full of people the next day who either think they have the condition or want to be screened for it. So these kinds of programmes, done often enough, will reach more and more people in the community and will reduce the level of ignorance about the disease in the community.

**What form of treatment is most suitable in your area: medication or surgery?**

The cost of eye drops is a major barrier against adherence to treatment in our patients. The cost of one month's treatment with the more effective drugs is more than the minimum wage in the country. Although there are cheaper generic medications on the market, they are not really available here.

We tend to recommend surgery as it is a single procedure. When successful, it maintains the pressure for a long time. However, surgery has its problems too, as the rate of failure of the filter is high in African patients and therefore regular follow-up is important. Another problem is that some doctors are reluctant to operate on patients if they have advanced glaucoma because of the risk of wiping out the remaining vision. If a patient does lose their vision after surgery, she or he may attribute that to the surgery, not the severity of the disease. Cases like these may give a clinic and the doctor a bad reputation and drive away patients.

Laser treatment is a good option where available. It is non-invasive, affords reasonable control over a long period of time, and can be repeated. Lately we have been offering diode laser cyclo-photo-ablation – one of the various laser procedures available for glaucoma – and our patients are accepting this more and more as an alternative to surgery and eye drops. The outcome of treatment has been good so far in the group of patients we are following.

**How difficult is it to ensure that people come for treatment and follow-up?**

Distance to glaucoma clinics is one of the reasons that stops patients coming to hospital. One way to remove this barrier would be to establish outreach clinics in communities, staffed by nurses and allied eye care personnel able to recognise glaucoma and refer patients.

When we investigated why some people did not come for follow-up visits, many patients we spoke to said they were put off by the unsympathetic attitudes of health care workers. They also complained about the long waiting times before being seen, clinic workers’ inability to retrieve their records, and queue-jumping by some patients.

**How can we improve patients’ experience?**

We must ensure that clinics are organised and that we treat patients on a strictly first-come, first-served basis. We must improve our record-keeping and filing systems, and reduce waiting times (see *Community Eye Health Journal* Issue 78: ‘Putting patients at the centre of eye care’ and *Community Eye Health Journal* Issue 74: ‘Ten years to VISION 2020: Why information matters’).

Our communication with patients must also improve. Many patients come to the hospital and receive treatment without knowing what they are being treated for. Health workers must explain patients’ diagnoses to them, say what glaucoma is all about, how it will impact on their sight and life, and discuss the management options with the patients (see page 54).

**‘Health workers must explain patients’ diagnosis to them, say what glaucoma is all about’**

Other factors that are barriers to treatment – such as the availability of facilities and drugs for treatment, the distance to such facilities, and availability and cost of drugs – must also be addressed.

**For those who are able to afford eye drops, what are the challenges?**

Patients sometimes stop using their eye drops because there is usually no improvement in vision and no relief of symptoms (as they had no other symptoms to start with). Therefore, there is little to motivate them to persist with their treatment. Continuing deterioration in vision is an issue even in patients who continue to use their medication as prescribed. When this happens, patients feel that their treatment has failed and they visit traditional healers instead of coming back to the clinic to find out about alternatives, such as surgery.

Traditional healers are often allowed to advertise on television and on local radio, and actively encourage patients to stop treatment. By the time patients come back to us, they are irreversibly blind, with no light perception, after having tried and lost faith in the traditional healers. They come back to us broke, despondent, and desperate for anything to restore their sight.

**What can eye care practitioners do to help?**

To ensure patients continue with their treatment, we must ensure that they know the following:

1. Their vision may not improve even when the treatment is effective.
2. There may be slight deterioration in their vision over time even when on medication as the treatment is not a cure but a control to delay blindness for as long as possible.
3. Patients should continue with their treatment even if the eye is completely blind. Doing so will prevent them from having a painful blind eye or a discoloured eye from corneal decompensation.

It is important to use strategies that motivate, empower, and commit the patient to change their behavior and improve their control of the glaucoma. We are exploring the use of motivational interviewing, administered by trained counsellors, to achieve this.

**Gender and glaucoma**

**Figure:**
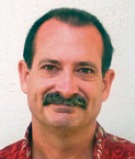
**Paul Courtright** Co-director, Kilimanjaro Centre for Community Ophthalmology, www.kcco.net

Evidence suggests that women account for about:

- 55% of people with open-angle glaucoma
- 70% of people with angle-closure glaucoma.

There are very few studies on service provision.

- In Tanzania, women account for a smaller proportion of people receiving surgical intervention for glaucoma.
- In Malawi, women who are suspected of having glaucoma are less likely to be referred to secondary facilities for assessment.

**What can we do?**

- Hospitals should be monitoring the use of medical and surgical services by for men and women separately. If you note more men receiving services than women, you should investigate.
- Screening programmes are rare; where they exist, findings for men and women should be assessed separately. Use the findings to revise the programme.
- Qualitative research is needed in a number of settings to understand any differences in how men and women with glaucoma seek help, whether medical or surgical. Use this information to develop gender-specific educational materials and programmes.
- In many settings, coming to hospital or clinic is difficult for women. Programmes should aim to create a more dynamic network to enable follow-up.

**Further reading**

Cook C, Foster P. Epidemiology of glaucoma: what's new? Can J Ophthalmology 2012; 47: 223–226.

Vajarant TS, Nayak S, Wilensky JT, Joslin CE. Gender and glaucoma: what we know and what we need to know. Current Opinion Ophthalmology 2010; 21: 91–99.

Mafwiri M, Bowman RJ, Wood M, Kabiru J. Primary open-angle glaucoma presentation at a tertiary unit in Africa; intraocular pressure levels and vision status. Ophthalmic Epidemiol 2005; 12:299–302.

